# Influence of spatial structure on protein damage susceptibility: a bioinformatics approach

**DOI:** 10.1038/s41598-021-84061-8

**Published:** 2021-03-02

**Authors:** Maximilian Fichtner, Stefan Schuster, Heiko Stark

**Affiliations:** 1 Department of Bioinformatics, Matthias Schleiden Institute, Friedrich Schiller University Jena, Ernst-Abbe-Platz 2, 07743 Jena, Germany; 2 Institute of Zoology and Evolutionary Research, Friedrich Schiller University Jena, Erbertstraße 1, 07743 Jena, Germany

**Keywords:** Biochemistry, Molecular biology, Systems biology

## Abstract

Aging research is a very popular field of research in which the deterioration or decline of various physiological features is studied. Here we consider the molecular level, which can also have effects on the macroscopic level. The proteinogenic amino acids differ in their susceptibilities to non-enzymatic modification. Some of these modifications can lead to protein damage and thus can affect the form and function of proteins. For this, it is important to know the distribution of amino acids between the protein shell/surface and the core. This was investigated in this study for all known structures of peptides and proteins available in the PDB. As a result, it is shown that the shell contains less susceptible amino acids than the core with the exception of thermophilic organisms. Furthermore, proteins could be classified according to their susceptibility. This can then be used in applications such as phylogeny, aging research, molecular medicine, and synthetic biology.

## Introduction

Aging research is a timely field of research that focuses on macroscopic and microscopic alterations during aging. Aging is a biological process in which a functional state is gradually declining or deteriorating^1–3^. Nevertheless, it is worth mentioning that aging is characterized not only by a decline in function but also by a remarkable robustness of many features such as the hematocrit value^4^, body temperature, and overall immune memory, etc.^5^. Macroscopic changes can be skin aging, reduced mobility, and organ damage (heart failure, autoimmune diseases such as age-related macular degeneration, neurodegenerative diseases such as Alzheimer's disease)^6–8^. At the molecular level, the non-enzymatic modifications alter proteins. This can lead to impairment in form, function, and/or degradability, which is then referred to as protein damage. The changes of the molecules are of decisive importance as they can accumulate in an organism and lead to macroscopic changes (e.g. the age pigment lipofuscin in the skin^9^). For many molecules, there are already detailed studies available. Well-known causes for misfolded proteins are mutation and repeat prolongation. Examples are provided by α-synuclein^10^, cystic fibrosis transmembrane conductance regulator^11^, peripheral myelin protein 22^12^, huntingtin (Htt)^13^, and Down syndrome critical region 1^14^. Here we present a computational study on the susceptibility of proteins to non-enzymatic modification rather than mutation.

### Score based estimation of peptide and protein susceptibilities

The present study extends our previous work, in which score tables were assembled and an approach to quantify peptide and protein susceptibility was proposed^15^. For the score tables known protein modifications from four literature sources were selected^16–19^. As an example, the hydroxylation of leucine, proline, and tryptophan could be mentioned here. On this basis, susceptibilities for the 20 standard amino acids (AAs) were determined. In a second step, the susceptibilities were weighted by text mining. This means that a search engine was used to find the respective amino acid with the respective modification in the corresponding database and the number of hits was counted. In a third step, a weighting was applied only with text mining and without the consideration of the modification table. Finally, an average of all scores was calculated. At first, this was applied without consideration of the three-dimensional (3D) structure.

In the present paper, we take the 3D structure fully into account. However, it can be expected that many peptides have no core in their spatial structures due to their short length. Nevertheless, we include them separately in our analysis since some of them do have a core such as insulin^20^. For simplification, only if necessary we make a terminological distinction between peptides and proteins, otherwise, we just use the term proteins for both in the following.

A similar approach in terms of structure scoring that connects well with our research has recently been published. There, the authors score the structures of proteins for their susceptibility to aggregate and call it AggScore^21^. An additional study (review) addressed AAs in the context of oxidation, which also ranked highest in our score (cysteine, tyrosine, and tryptophan)^22^. They also mention the problem in connection with the storage of biotherapeutics for longer durations. It is notable that some other AAs mentioned in that study^22^ like histidine, methionine, and phenylalanine were just ranked average in our score^15^.

### Spatial protein structures

In contrast to our previous work, which mainly considered the primary structure (complete amino acid chain)^15^, here we take spatial information into account. Obviously, the susceptibility of an AA to spontaneous modification depends on its localization within the protein. We make a terminological distinction between ‘shell’ and ‘surface’ in that the shell is a layer of amino acids and is three-dimensional while the surface is the two-dimensional outer boundary of the shell. AAs in the protein shell are much more easily accessible to, for example, reactive oxygen species than those in the core. In addition to complete proteins, we also characterized specific secondary structures^23–26^ (Suppl. Table S2). This might be relevant for the susceptibility of proteins during the folding process.

Within the tertiary structure, the composition of secondary structures is decisive for the accessibility of the AAs. If, for example, there are more α-helices outside than β-sheet structures, a different impact on the shell/surface is to be expected. In the calculation of the whole protein shell, this issue is already addressed.

Furthermore, the surface can be covered by other structures. In the quaternary structure, individual proteins form protein complexes. After the formation of a protein complex, a new protein surface is formed. Another example would be proteins that can be embedded in membranes. Thus the protein is only partially accessible to the respective micro-environments (e.g. mitochondria, chloroplasts, …)^27^. Especially for enzymes, susceptible/functional areas can also be hidden in pockets^28^. However, here we focus only on the tertiary and possibly also the quaternary structure since this represents the final form of the proteins. It should be noted that the spatial structure is subject to fluctuations and this can lead to differently measured data^29^.

### 3D approach

The idea is to establish a 3D approach where only the AAs which are lying in the protein shell are considered for the calculation. There are a number of algorithms for the calculation of protein geometries, which calculate the protein surface, volumes, and pockets^29–33^. For example, Li et al. could calculate the surface (alpha shape) with the help of the Delaunay triangulation^33^. Here the outer AAs need to be identified in such a way, yet for the whole shell. In contrast, the remaining AAs form the protein core. With this method, it is possible to calculate different susceptibilities between shell and core. This method has a lower spatial resolution than the approaches cited above but has the advantage of being faster.

Additionally, in the susceptibility analysis, we make a distinction between the backbone and the side chains. Depending on the folding, the respective parts are accessible from the outside.

### Hypothesis

Compared to our earlier results^15^, it can be expected that there will be a difference between the whole and parts of the protein. The AAs in the core are protected by the protein shell and could have more susceptible AAs. On the other hand, we expect the AAs in the shell to be more selected towards robustness. On closer inspection, the shell can also be analysed with regard to its differences with and without backbone and with and without side chains.

## Results

### Raw data

The first step was to evaluate the raw data for the various analyses (Table 1). It should be noted that whole protein (WP) contains all proteins. The calculation for protein shell (PS) resulted in as many entries as WP (since all proteins have a shell). The AA count for 6 Å is larger than for 7 Å, since the penetration depth of the concave hull is larger so that more AAs are found (this applies to all calculated shells). With shell backbone (SBB) almost no proteins are lost compared to PS. However, when comparing the AA count of SBB with PS it is noticeable that fewer AAs are selected. With shell side chain (SSC) the number of proteins is reduced, i.e. there are proteins without an external side chain atom. In the 7 Å variant, this is even more pronounced because here the net has a lower density. When comparing the AA count of SBB with SSC it is noticeable that there are many more side chains outside than backbones. Not every protein has a core (PC). In many peptides and small proteins, all amino acids are accessible from the outside. That is why here the number of proteins is lower compared to WP. Note that these terms (PC, WP,…) are based on a mathematical approach and are not derived from a biological terminology.

**Table 1:** Results of the number of proteins of the respective analysis (*WP* whole protein, *PC* protein core, *PS* protein shell, *SSC* surface side-chain, *SBB* surface backbone) and amino acid(AA) count.

Peptides/proteins	Cavity 6 Å–count [AA count]	Cavity 7 Å–count [AA count]
WP	131,458 [94,411,474]	
PC	129,480 [57,626,370]	129,688 [60,348,977]
PS	131,458 [36,785,104]	131,458 [34,062,497]
SSC	131,092 [31,659,172]	131,092 [29,389,352]
SBB	131,434 [17,328,586]	131,432 [14,588,173]

### Comparison of linear versus spatial approach

**Figure 1:**
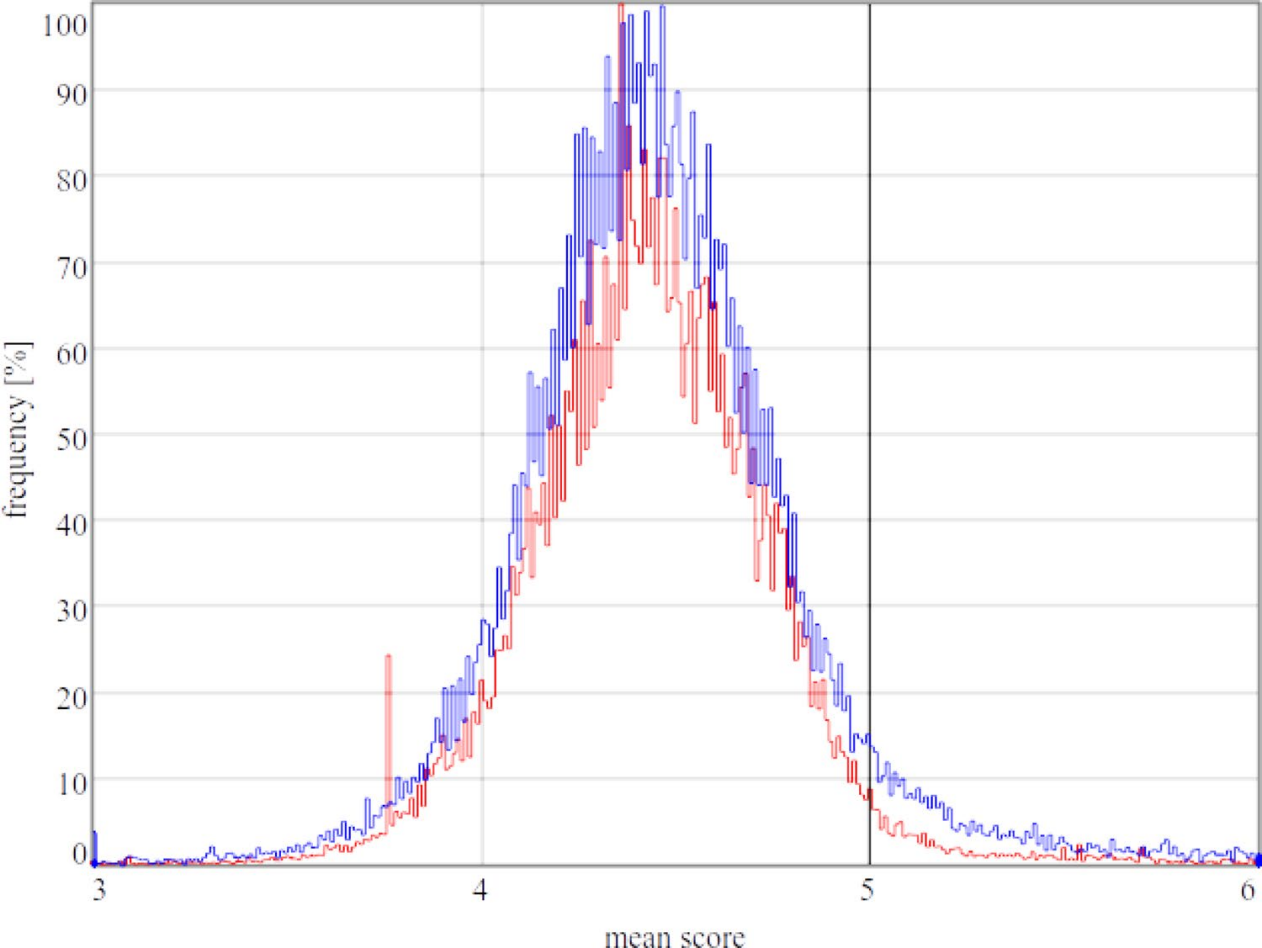
Comparison between the normalized histogram computed in our earlier paper^15^ (blue) and the normalized histogram for the spatial approach (red). The red peak on the left (~ 3.757) is mainly due to multiple entries of endothiapepsin of the organism *Cryphonectria parasitica* in the data set. The second and highest peak (~ 4.357) is mainly due to lysozyme of the organism *Gallus gallus*^28^. The black line indicates the mean score of a random artificial protein. Graphic created with Enzyme2 version 9.7.22 (https://starkrats.de).

**Figure 2:**
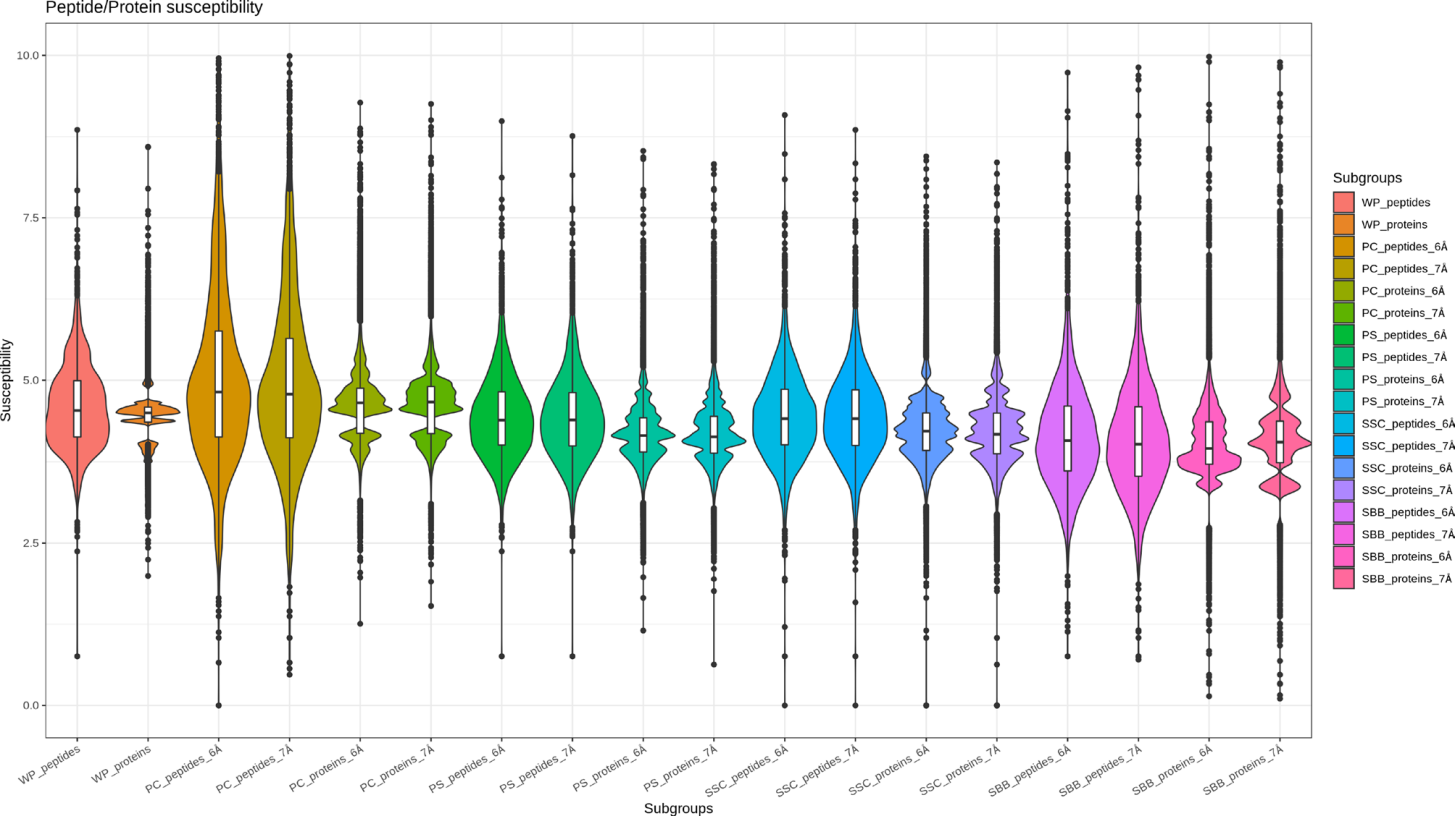
Violin- and boxplot of the influence of the spatial peptide and protein structures (*WP* whole protein, *PC* protein core, *PS* protein shell, *SSC* shell side chain, *SBB* shell backbone) on the susceptibility of peptides and proteins for a cavity of 6 Å and 7 Å. The violinplot is normalized to the number of every subgroup. Created with R version 4.0.3 with packages tidyverse and ggplot2.

The amount of molecules (131,458) is smaller compared to that analysed earlier^15^ (422,091) since the spatial structure is not known for each molecule. A direct comparison of the histograms between these two studies shows a good correlation with regard to the normal distribution (Fig. 1). A further comparison of the distribution with respect to PS, SSC, SBB, and PC shows that the normal distribution is shifting (Fig. 2; Suppl. Tables S3, S4). The SBB has the lowest mean and PC the highest. PS, SSC, and WP are, in that order, situated in between. Our analysis showed that the choice of the AAs belonging to the PS is decisive for the way the susceptibility is calculated. There is a difference between 6 or 7 Å cavities (Suppl. Tables S3, S4). The peptides show a higher standard deviation (SD) and lower mean values compared to the proteins.

### Validation of amino acid distribution in the protein shell

**Figure 3:**
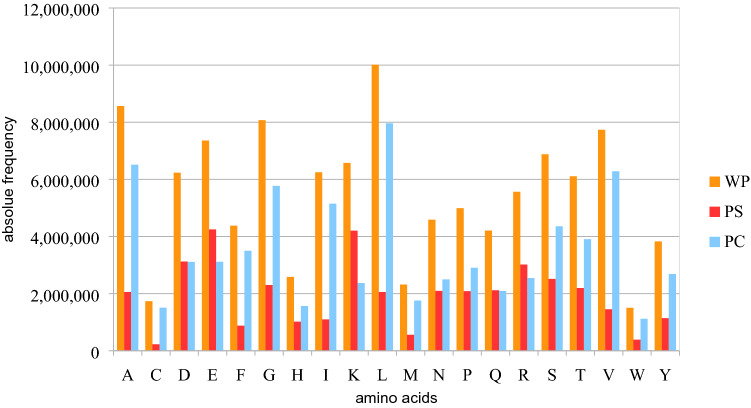
Absolute frequencies of the 20 standard amino acids in the data and their distribution over protein shell (PS) and protein core (PC). The blue lines under the letters mark the hydrophobic amino acids. For further analysis see Suppl. 1 and 7. Diagram created with LibreOffice Calc version 6.4.6.2.

It is well known that, in cytosolic proteins, hydrophilic AAs are mainly found in the shell and hydrophobic AAs in the core^10^. These findings agree with our results and show that our algorithm correctly discriminates against the shell (Fig. 3; further analysis see Suppl. Figs. S1, S7). Notice that in our data set the PC contains 64.3% and the PS 35.7% of all the AAs. The basic AAs lysine (K) and arginine (R), as well as the acidic AA glutamic acid (E) are hydrophilic and stand out due to a higher proportion in the PS. It should be mentioned here that K, R, and E have a medium susceptibility, with E having the lowest value^15^. Furthermore, the AAs glutamine (Q) and aspartic acid (D) show an equal distribution between PS and PC. In contrast to K, R, and E, however, they show the lowest susceptibility. In addition, it is shown that the most susceptible AAs, i.e. tyrosine (Y), cysteine (C), tryptophan (W), and leucine (L), are represented above average in the core.

### Comparison of protein shell and protein core

**Figure 4:**
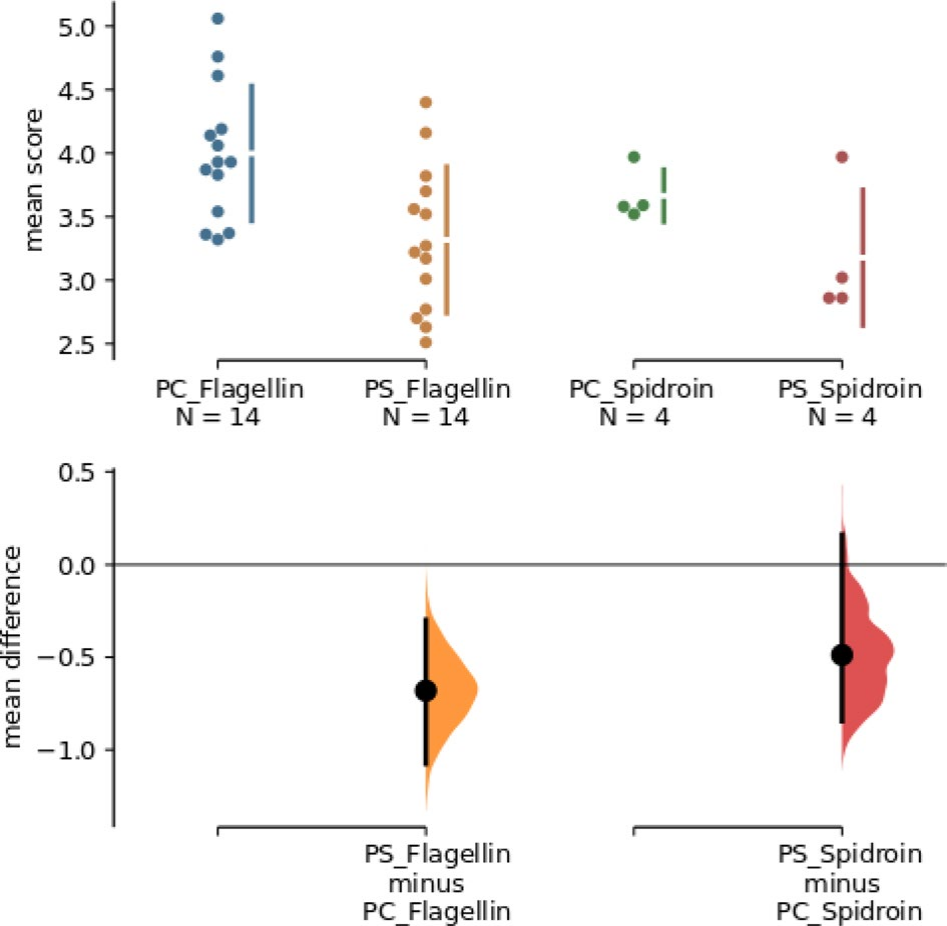
Comparison of mean differences of the protein core (PC) and protein shell (PS) for the flagellin and spidroin protein shown in a Cumming estimation plot. The raw data is plotted on the upper graph. Mean differences are depicted as dots; 95% confidence intervals are indicated by the ends of the vertical error bars. Each mean difference is plotted on the lower graph as a bootstrap sampling distribution (5,000 bootstrap samples; confidence interval is bias-corrected and accelerated). Graphic created with estimation statistics (https://www.estimationstats.com)^54^.

In the previous paper, reduced susceptibilities were shown for some proteins, among others for flagellin and spidroin^15^. A more precise differentiation between PS and PC shows that the shell of these proteins is less susceptible than the core and thus appears in the lowest susceptibility score for PS (Fig. 4; flagellin P-value = 0.00471; spidroin P-value = 0.243). For further analysis see Suppl. 8.

**Figure 5:**
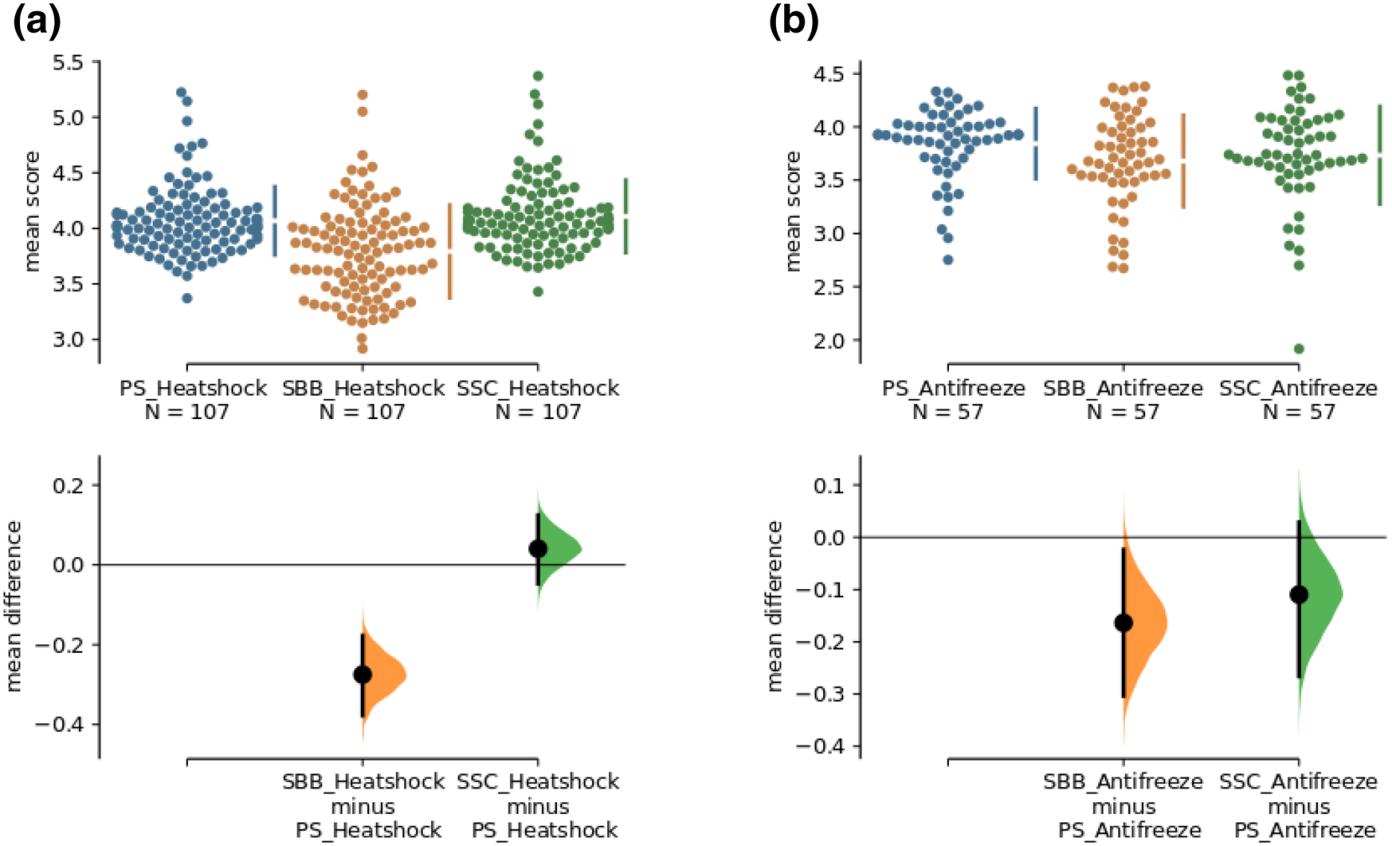
(**a**,**b**) Comparison of mean differences of the shell (PS, SBB, SCC) for the heatshock and antifreeze protein shown in a Cumming estimation plot. The raw data is plotted on the upper graph. Each mean difference is depicted as a dot. Each 95% confidence interval is indicated by the ends of the vertical error bars. On the lower graph, mean differences are plotted as bootstrap sampling distributions (5,000 bootstrap samples; confidence interval is bias-corrected and accelerated). Graphic created with estimation statistics (https://www.estimationstats.com)^54^.

When analyzing the shell, the location of the backbone or side chain is important for the analysis. For example, the heatshock protein shows that the proteins significantly influence the susceptibility calculation for the backbone (Fig. 5; SBB-PS P-value 7.1e-08; SCC-PS P-value 0.327). However, this does not apply to all proteins. For the antifreeze protein, it can be shown that there are only minor differences between these different approaches (Fig. 5; SBB-PS P-value 0.0123; SCC-PS P-value 0.118).

### Comparison of specific protein groups

As shown above, our method is suitable to study differences in spatial modification susceptibility within certain protein groups. We have shown this with the example of hydrolases, lyases, and isomerases (Suppl. 9). A mean shift of the susceptibility becomes visible. Furthermore, it is possible to compare proteins via groups of organisms. We have investigated this for the genera Sulfolobus, Escherichia, Arabidopsis, Saccharomyces, Drosophila, and Homo. We could find clearly visible differences between unicellular and multicellular organisms (Suppl. 10, Suppl. Fig. S10). In particular, we found a difference between thermophilic and non-thermophilic organisms (Suppl. 10, Suppl. Fig. S11). A detailed list of additional groups can be found on the website http://damage.stark-jena.de.

## Discussion

Here we have analysed 131,458 peptides and proteins from the PDB, to provide an initial pool for further studies. By way of example, we selected specific subsets of proteins such as hydrolases, lyases, isomerases and heat shock proteins. However, the main goal here was to establish a scoring scheme including structural information rather than an in-depth analysis of specific protein families.

### Protein core and protein shell properties

**Figure 6:**
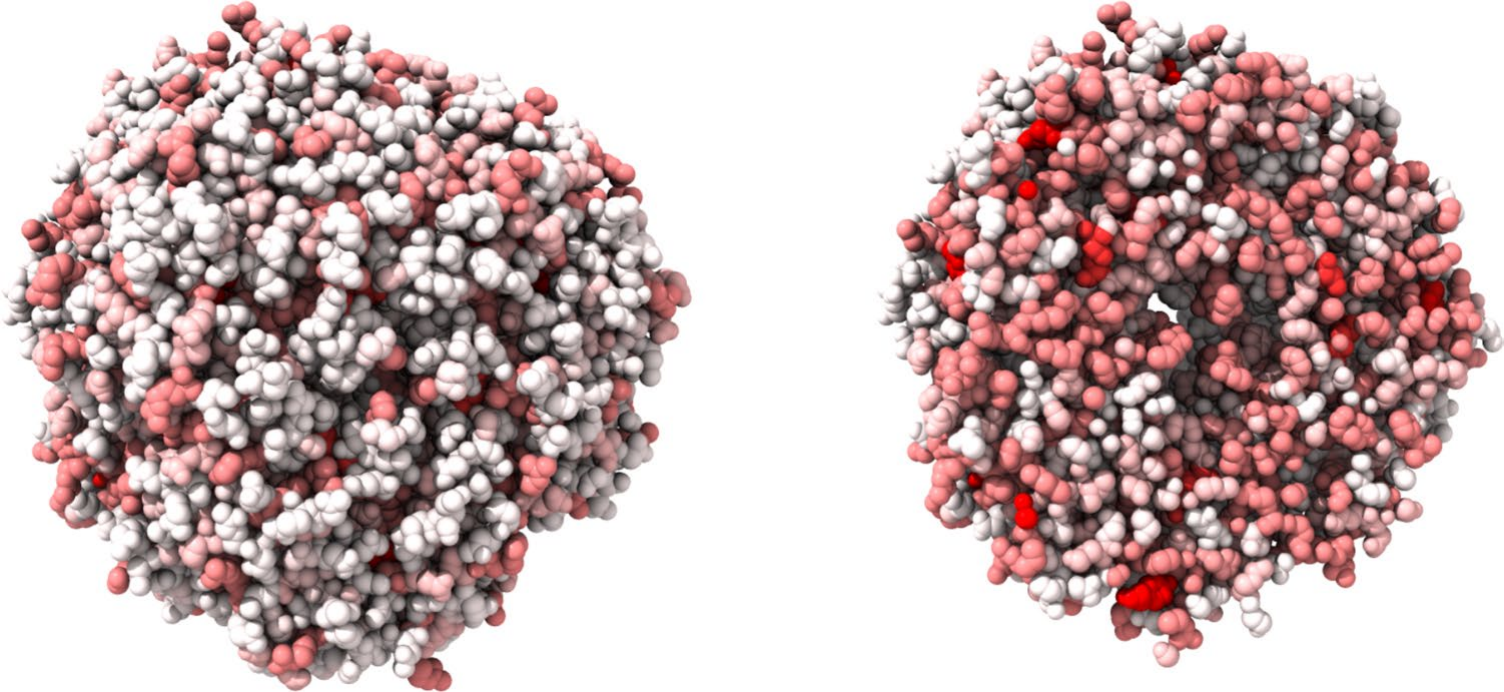
Protein almond Pru1 (PDB entry: pdb3fz3). Left: view on the whole protein, right: section view. That protein has a strong diversity between the shell (low susceptibility—white) and core (high susceptibility—red). Graphic created with UCSF ChimeraX version: 0.91^55^.

**Figure 7:**
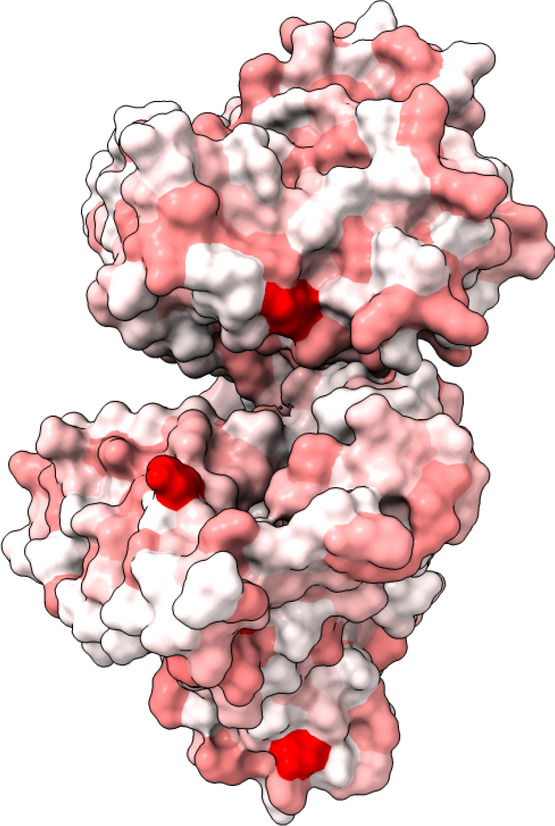
Shell/surface susceptibility of the protein phosphodiesterase 4B (PDB entry: pdb5ohj). Graphic created with UCSF ChimeraX version: 0.91^55^.

Our hypothesis was that protein shell (PS) involves evolutionarily less susceptible AAs than the protein core (PC) because it interacts more with the environment (Fig. 6). We could confirm this hypothesis for the mean values of most proteins (see ‘Results’). However, there are some exceptions with regard to the sorting by organisms (see next subsection). It should be noted that the organisms (unicellular versus multicellular) may be exposed to different environments. However, there are proteins that differ from our hypothesis and for which the surface is important for protein interaction (Fig. 7)^34^. This can lead to a change in the susceptibility of the PS. This is desirable, for example, in order to subsequently industrially modify proteins^35^.

It is also known that proteins with susceptible AAs (tyrosine) in the shell are relevant for biological aging and age-related disease^36^. On the other hand, certain AAs protect proteins. For example methionine, which has antioxidant and repair properties^37–39^. The proteostasis theory in aging research is based on the assumption that deficiencies in natural protein turnover, such as misfolded proteins and deleterious protein aggregations, are a major cause for a decline in cell function^2^. One important phenomenon in this context is the non-enzymatic, modification of amino acids within proteins. By our calculations, predictions can be made on how susceptible particular proteins are to this progressive decline in protein homeostasis. Moreover, it is obvious that the decline can be delayed if highly susceptible amino acids are buried in the core of proteins. This is supported by our results, except for thermophilic organisms.

An important argument concerning the PS is how far it lies in or on membranes and how much it is protected by them. For example, the proteins of the respiratory chain are embedded in membranes while one side is in contact with a more aggressive environment^40^. It is well known that many proteins in the intermembrane space (IMS) contain conserved cysteine-rich sections^41^. There is still no explanation for the function of these tracts^41^. It should be noted that according to our previous results, the second most susceptible AA is cysteine ^15^. Specific information on membranes could not be taken into account by our approach because the exact location of every protein was not in the database. The same applies to complexes (if they were not contained in the database), which can protect parts of the protein shell/surface from modification.

A comparison of the results on the basis of the different distance values (6 Å or 7 Å) shows that the mean susceptibility value is similar (Suppl. Tables S3–S6). Thus the method is not very sensitive to the choice of this parameter value. The distinct difference between shell and core remains unaffected.

### Further properties—enzymes

The distinction between protein shell and core is particularly important for enzymes since the active site is usually hidden in pockets and is either presented by a conformational change or accessible by the key-lock principle^28^. Here no distinction is made between enzymes and non-enzyme proteins and thus the active sites are not examined in detail. But these hidden pockets can be an advantage in addition to substrate specificity in terms of avoiding modifications. One example is the ALDH enzyme, where the oxidation of a specific AA (Cys302) inactivates the enzyme^37^. In that paper, it was shown that neighbouring cysteine AAs protect the catalytic cysteine by covalent bonds.

When it comes to protein damage, a loss of functionality does not necessarily imply a heavy damage leading to aggregation. The required modifications for that purpose are very variable. On one hand, nearly every modification leads to a loss of function due to changes in the conformation or in the functional domain^18^. For this, the active and passive forms would have to be taken into account. On the other hand, there is an earlier finding of multiple methionine modifications not leading to a loss of function^38^. Altogether we suspect that functionality has had higher priority compared to robustness in evolution.

### Phylogeny specific issues

The analysis, taking into account the organisms revealed a differentiation with regard to susceptibility, which could be due to the multicellularity and the adaptation to the environment (Suppl. Fig. S10). As the analysis of the PS and PC data suggested, the environment for unicellular organisms showed a shift of shell/core susceptibilities. For the individual consideration of the organisms, a differentiated picture may occur. For example, in S*ulfolobus* against our hypothesis the PS is more susceptible than PC in contrast to most of the other organisms (Suppl. Fig. S11). The genus S*ulfolobus* is characterized by optimal growth rate at pH 2–3 and temperatures of 70–75 °C^42^. While it keeps a pH-value of 6.5 in the cytosol^43^, the high temperature could be related with the more susceptible PS. This susceptibility pattern also applies in the attenuated form to all other unicellular organisms which, in contrast to multicellular organisms, have a higher reproduction rate. This allows them to get rid of damaged proteins by asymmetric division or apoptosis. Multicellular organisms, on the other hand, do the same, but the damaged proteins remain in the intercellular spaces or have to be expensively transported away (immune cells^44,45^). We hypothesize that the outer cell layers (e.g. a major constituent of the skin is collagen) of multicellular organisms have a typical proteome that protects them from an aggressive environment.

### Caveats

The drawbacks of the approach leading to the score that was used in this work, were already discussed in our previous paper^15^. The following limitations of the spatial approach are worth mentioning. In this work, it was only possible to analyse conformational changes if different conformations of the same protein are stored in different entries (only one per file) of PDB. However, with some modification to the approach, this would become feasible.

Moreover, the value of the distance parameter is crucial for the outcome. This leaves some room for variability. A value smaller than 6 Å is, however, not reasonable in this context. Further details are described in the ‘Methods’ section.

Here we only look at the mean susceptibility score of the protein shell in terms of non-enzymatic modification. There seem to be examples where specific AAs are very prominently modified^38^. This leads to the question: what if one or a few specific AAs in the shell have more impact in terms of susceptibility than the average mean? This would lead to some follow-up questions. What AAs are basically affected by this? Where are they generally positioned in the shell? Is this possibly connected with repair, signalling, or other mechanisms? In order to analyse specific AA positions in proteins, some adjustments to the approach would be necessary. This is out of the scope of this work and an interesting point for future studies.

### Possible extensions

In this paper, we have investigated the susceptibility of AAs in proteins with respect to their spatial location. A further interesting classification model is quantitative structure-activity relation (QSAR) analysis^46,47^. Here, in contrast to our analysis, a relationship is established between the structure and the activity. This can also be combined with our classification model relating structure and aging (susceptibility).

Another approach would be to describe the exact spatial orientation of the AA susceptibilities using a tensor. The different proteins could be sorted according to linear, planar, or spherical spatial susceptibility. It is to be expected that e.g. membrane proteins, which pass through the membrane, could show a linear part and surface-associated proteins show rather planar properties. Unbound proteins are more likely to have spherical susceptibilities because they can be attacked from all sides. In future studies, sequence-based methods for predicting the location or the formation of protein complexes could be included.

A further approach would be to study the accessibility on the atomic level in the form of a spherical representation with a calculated surface fraction called relative solvent accessible surface area (RASA)^30,48^. This is a possible extension of our work since the surface fractions can be used as weightings and the single atoms can be scored in terms of their susceptibility. For reasons of simplification, however, we have limited ourselves to the AAs. This has the advantage that the inaccuracy of the spatial structure has less influence on the weighting. Inaccuracies can be e.g. the degrees of freedom of individual AAs, as well as the free energy, volume, or entropy. An example is provided by the transcription factors (some of them involve many random coils), which often have an undefined surface and are therefore difficult to calculate.

A future application concerns the use of susceptibility scores in dynamic simulation of proteins. This could be analysed in a similar way as was done here for the structural changes to the protein superoxide dismutase 3 in the case of several missense mutations^49^. Additionally, the study could be extended to look also into other parameters like surface hydrophobicity or, in our case, the (shell/surface) susceptibility to non-enzymatic modification. An important benefit for synthetic biology is the knowledge about the susceptibility of proteins to oxidation in connection with storage and the associated degradation^22^. In synthetic biology also non-proteinogenic AAs are relevant. The approach proposed earlier^15^ can be extended to those AAs. If no data are available from text-mining, a first approximation is to use the susceptibility value for structurally related proteinogenic AAs, such as leucine for norleucine.

Many extensions are conceivable in aging research. From the calculated scores of proteins, predictions can be derived for the impact on aging processes. This may apply to specific proteins that have not been investigated so far in the context of aging.

## Methods

### Preprocessing

As a basis for the analysis, the spatial structures of all molecules (139,291) deposited in the Protein Data Bank (PDB) were downloaded (see Suppl. 3). In the preprocessing, only the compositions which contain AAs in their sequence were considered (Suppl. 6). In addition, the data sets may include also ligands and water. These were retained for the calculation if provided, as they form the outermost shell and can act as protection. In future studies, it would be worth comparing all the calculations with or without water and ligands. Here, we have performed an example comparison for the protein human oxyhaemoglobin (PDB: 1hho; Suppl. 3).

A direct comparison for multiple spatial structures is too challenging due to combinatorial explosion (comparing all atoms with each other), that is why only one spatial structure per entry was considered (Suppl. 3). This means every file with multiple structures has been excluded. Additionally, the entries with redundant sequences may differ in their spatial structure, resulting in different shell and core compositions, and were therefore not summarized here. For our analysis we concentrate only on the PDB data. A connection with further databases would be possible. For example to investigate the influence of domains.

### Whole protein

We have developed nine different approaches to compare the influence of the spatial structure. The simplest one, ‘whole protein’ (WP) considers all AAs (like the theoretical approach^15^). Based on these results, we again calculated the susceptibilities for all proteins for which we had the spatial data (Suppl. 3). For that purpose, we only used the score ALL of the previous paper^15^. This allows us to compare parts (shell, core) with the whole protein in terms of susceptibility. Score ALL is a mean of six scores with different focuses in terms of weighting and database. Using the mean value, it was tried to combine the best characteristics of the scores.

### Protein core and protein shell

By defining the protein parts, we can mainly examine the ‘protein core’ (PC) on the one hand and the ‘protein shell’ (PS) on the other. The outer surface of the PS can be interpreted as the solvent accessible surface area (SASA/ASA), although this excludes the surface of interior holes. Accordingly, the classification proposed here (PC, PS) refers to a sequence of amino acids rather than a surface area. In addition, a distinction is made in the shell between ‘shell side chains’ (SSC) and ‘shell backbone’ (SBB) depending on which part of the AA points outwards and will therefore be accessible. Note that the backbone is almost the same for every AA in terms of structure and susceptibility. The score is heavily depending on the properties of the respective AA, which in turn is mainly based on the side chain structure. If only the backbone would point outwards, this would lead to a bias. That is why another approach was implemented where the whole shell is analysed on the atomic level and then the AA with only backbone atoms pointing outwards are excluded from the analysis (SSC). For the sake of completeness, another approach was devised in which the AAs with only side chains pointing outwards are subsequently removed (SBB). Thus, it is possible to analyse the number of side chains of individual proteins in comparison to the backbone in the protein shell.

For the determination of the PS, SSC, SBB (biological terms) the concept of the ‘concave hull’ (mathematical term) is used. The algorithm described here was implemented in the program Cloud2 Version 14.3.20 (Heiko Stark, Jena, Germany, URL: https://starkrats.de). Cloud2 was originally designed for spatial data analysis and was used for the differentiation of the different protein parts (Suppl. 3).

Starting from the convex hull, the concave hull can be calculated with a termination criterion. For the convex hull there are various algorithms with different runtimes (Gift wrapping, Graham Scan, Quickhull, Divide and conquer, …). Not all of the 2-dimensional calculation algorithms can be generalized to the 3-dimensional case. The Delaunay triangulation is worth mentioning, which can be generalized to that case and was used by Li et al.^33^.

In the present work, we use an extended version of the Graham Scan with a termination criterion for the definition of our PS^50^. With the algorithm, the convex hull of a finite set of points in 2D can be calculated. After that, we check which line connecting two atoms in the hull is longer than the minimal distance (6 Å or 7 Å). If so, the line is replaced by two or more lines connecting the two atoms with the closest indented node(s). Finally, this results in the concave hull. This simplification is generalized to sectional planes along the three spatial axes to identify all surface-associated atoms. In this way, we obtain the concave hull, but only for topologies with genus zero (i.e. without holes). The results of this method are very similar to those of the approach proposed by Lee and Richards^30^ in which a probe sphere rolls over a van der Waals surface. Effectively our method gives the same results using a different perspective.

The calculation of the concave hull is a complex problem in information theory. There is no agreed definition for it since a decision is necessary at which point the algorithm should stop chasing deeper gaps. That is why the concave protein surface will be defined depending on the requirements of the problem in question. For this purpose, we chose a small radical (hydroxyl radical), which is reactive at any pH value. With the help of the software ‘Avogadro’ Version 1.1.1^51,52^ we determined the minimum distance for chasing deeper gaps. While the Van-der-Waals radii of bound proteinogenic atoms are larger, the radii of isolated atoms are between 1.1 and 1.8 Å ^20,53^. Here an oxygen radical (1.52 Å) was moved through two carbon atoms (1.7 Å) and the distance was measured (6–7 Å). These values are to be understood as the lowest limit where a radical may be able to interact with a deeper lying AA. Radicals come in different sizes and not every radical may be able to reach deeper sections of a protein. It should be noted that this is not a fixed limit and it is therefore only an approximation. A rough formula could be: X_D_ + 2 × ½ Y_D_ = X_D_ + Y_D_ where X_D_ is the Van-der-Waals diameter of the penetrating atom and Y_D_ is the Van-der-Waals diameter of the two surface atoms that are not bound with each other. The respective formula for radii would then be 2 X_R_ + 2 Y_R_. We used the two slightly different values of 6 Å and 7 Å to perform a sensitivity analysis. This allows us to show to what extent the results depend on the parameter value chosen.

**Figure 8:**
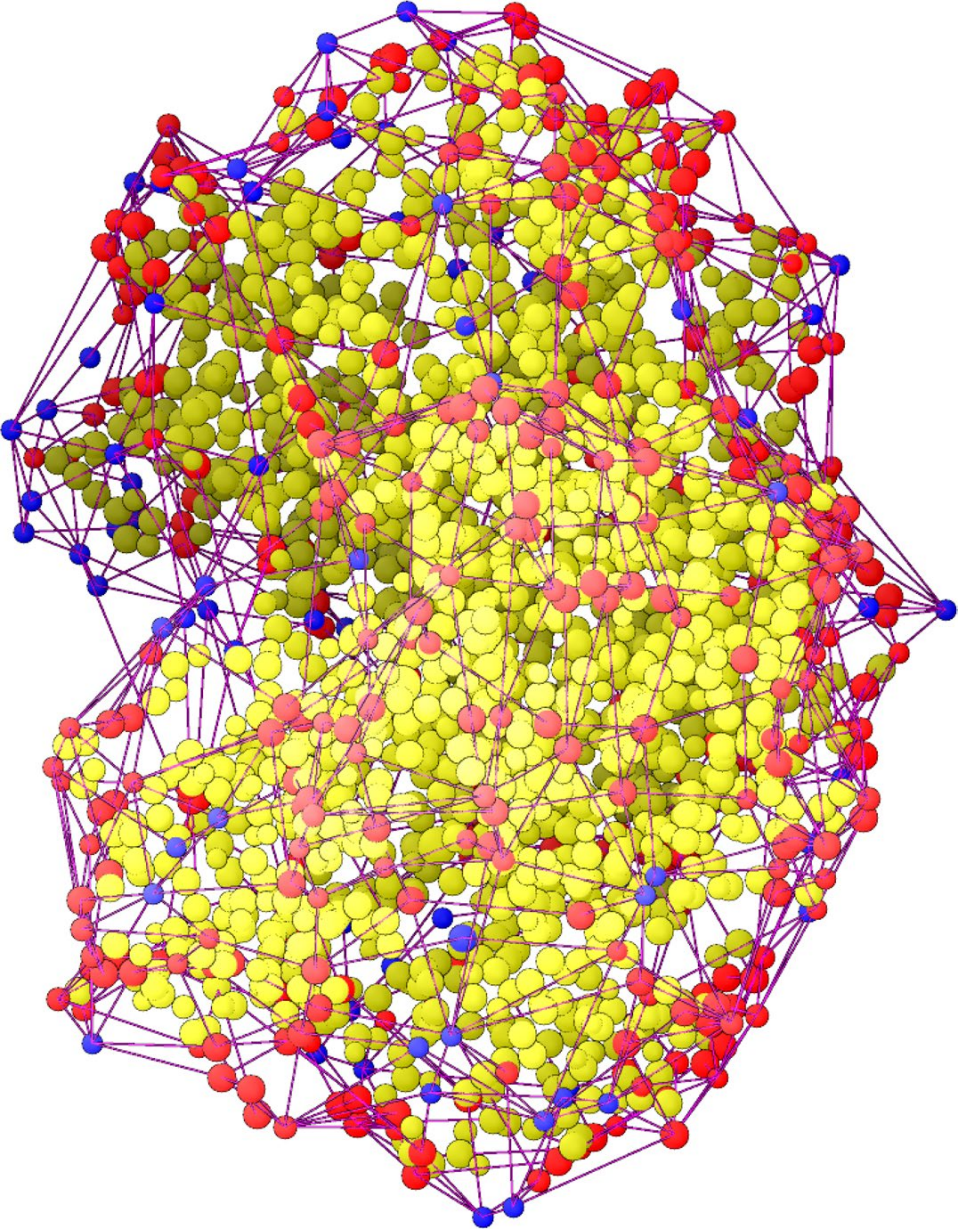
Representation of the amino acids and water/ligands components of the protein oxyhaemoglobin (PDB entry: pdb1hho). A dot represents the averaged centre of an amino acid (red when in the protein shell (PS), yellow in the protein core (PC)) or water molecule/ligands (blue). Red lines show the cross-linking by the surface calculation, by which the surface (concave hull) is defined. All unlinked dots are assigned to the PC. Graphic was with Cloud2 version 15.7.22 (https://starkrats.de).

The surface contains the coordinates of the external atoms of the molecule and in a second step, the corresponding AA to these atoms were determined (Fig. 8). After this assignment, shell and core of the protein can be separated.

### Scoring

As a first step, the entries with multiple conformations were removed for reasons of complexity. This included 8039 (5.77%) entries. From this data, an AA count was performed for validation. In addition, the sequences for the different protein parts (WP, PS, SBB, SSC, PC) were weighted with the score ALL and a modified scoring program (^15^; Suppl. 3). In a second step, the AA sequences with more than 5% X (unknown AAs) were removed (named with ‘X correction’; this makes 5.40% of all entries). The 5% were taken from the previous paper^15^.

### Classification

Based on the annotations in the protein lists, a classification of special protein groups (collagen, cytochromes, ribosomes, …), organisms, and enzymes was possible. This has been realized with the tool Enzyme2 Version 8.3.20 (Heiko Stark, Jena, Germany, URL: https://starkrats.de). For this purpose, the complete list was searched for specific patterns (e.g. enzymes: lyases, isomerases,… see Suppl. 9; organisms: human, mouse,… see Suppl. 10) and combined histogram-barcode plots were created.

## Supplementary Information

Supplementary Information 1.

## Data Availability

The datasets generated during and/or analysed during the current study along with the code and several supplementary files are available in the Mendeley repository: Fichtner, Maximilian; Schuster, Stefan; Stark, Heiko (2021), ‘Data for: Influence of spatial structure on protein damage susceptibility—A bioinformatics approach’, Mendeley Data, V1, doi: https://doi.org/10.17632/jkmbpfgp4k.1.

## Abbreviations

3D: Three-dimensional
AA: Amino acid
PC: Protein core
PDB: Protein Data Bank
PS: Protein shell
SBB: Shell backbone
SD: Standard deviation
SSC: Shell side chain
WP: Whole protein
