# Supplementary material for: Influence of spatial structure on protein damage susceptibility: a bioinformatics approach
Source: Sci Rep. 2021 Mar 2;11:4938. doi: 10.1038/s41598-021-84061-8 (PMC7925522; doi:10.1038/s41598-021-84061-8)
Supplement: Supplementary file 1 — Supplementary Information 1. [file 41598_2021_84061_MOESM1_ESM.pdf]

Supplementary Information for:

Influence of spatial structure on protein damage susceptibility – A bioinformatics approach

Maximilian Fichtner[1,*], Stefan Schuster[1], Heiko Stark[1,2]
[1]Matthias Schleiden Institute, Department of Bioinformatics, Friedrich Schiller University Jena, Ernst-Abbe-Platz 2, 07743 Jena, Germany
[2]Institute of Zoology and Evolutionary Research, Friedrich Schiller University Jena, Erbertstraße 1, 07737 Jena, Germany
*Corresponding author, email: maximilian.fichtner@uni-jena.de

Table of Contents | Page

1. Hydrophobicity classification S2
2. Spatial accessibility of secondary structures S3
3. Data preparation S4
4. Extended Graham Scan S6
5. Statistics of the influence of the spatial protein structure on the susceptibility S6
6. Example Comparison with and without water and ligands S7
7. Further amino acid distribution visualizations S8
8. Further comparison of protein shell and core properties S10
9. Further comparison of enzyme properties S12
10. Comparison between organisms S13
11. Top and Bottom 100 S14
12. Appendix S14
13. References S21

1. Hydrophobicity classification

We try to gather information about the amount of hydrophobic AAs for every protein to give a relative representation of the bonding potential of hydrophobic interactions that lie within the proteins. When it comes to conformational changes this can lead very easily to aggregations. Beware this information is not oriented on protein surface hydrophobicity! It is generally known that there is a difference in the distribution of hydrophobic versus hydrophilic AAs[1]. This is because proteins have an aqueous environment and therefore have to be hydrophilic.

Concerning hydrophobicity there is no definite classification. Here we are holding on to the description of Voet and Voet[2], Lesk[3] and Berg, Stryer and Tymoczko[4] with some minor adjustments because we are only interested in hydrophobic potential. That means all the nonpolar AAs which are alanine, phenylalanine, glycine, isoleucine, leucine, methionine, proline and valine are definitely classified as hydrophobic. Two other borderline cases namely tyrosine and tryptophan are also seen as hydrophobic due to their aromatic rings (Tab. 1).

Glycine for example has a side chain which is so small that the attributes of the backbone dominate. That means in unbound form it would be hydrophilic and when bound it would be hydrophobic. Since here we are working with bound AAs the classification is decided to be hydrophobic. A contradiction for the first and last AA in the chain will be neglected.

Suppl. Table 1: The upper table shows the properties of the amino acids with regard to hydrophobicity. The lower table shows the classifications of amino acids with regard to hydrophobicity in the literature. The representation of the bold entries show the own decisions about different views in the literature regarding hydrophobicity.

amino acid	A	C	D	E	F	G	H	I	K	L	M	N	P	Q	R	S	T	V	W	Y
hydrophobic	X				X	X		X		X	X		X					X	X	X
hydrophilic		X	X	X			X		X			X		X	X	X	X			X

amino acid	A	C	D	E	F	G	H	I	K	L	M	N	P	Q	R	S	T	V	W	Y
Voet & Voet	X				X	X		X		X	X		X					X	X	
Lesk	X				X	X		X		X	X		X					X		
Stryer	X				X	X		X		X	X		X					X	X	
This work	X				X	X		X		X	X		X					X	**X**	**X**

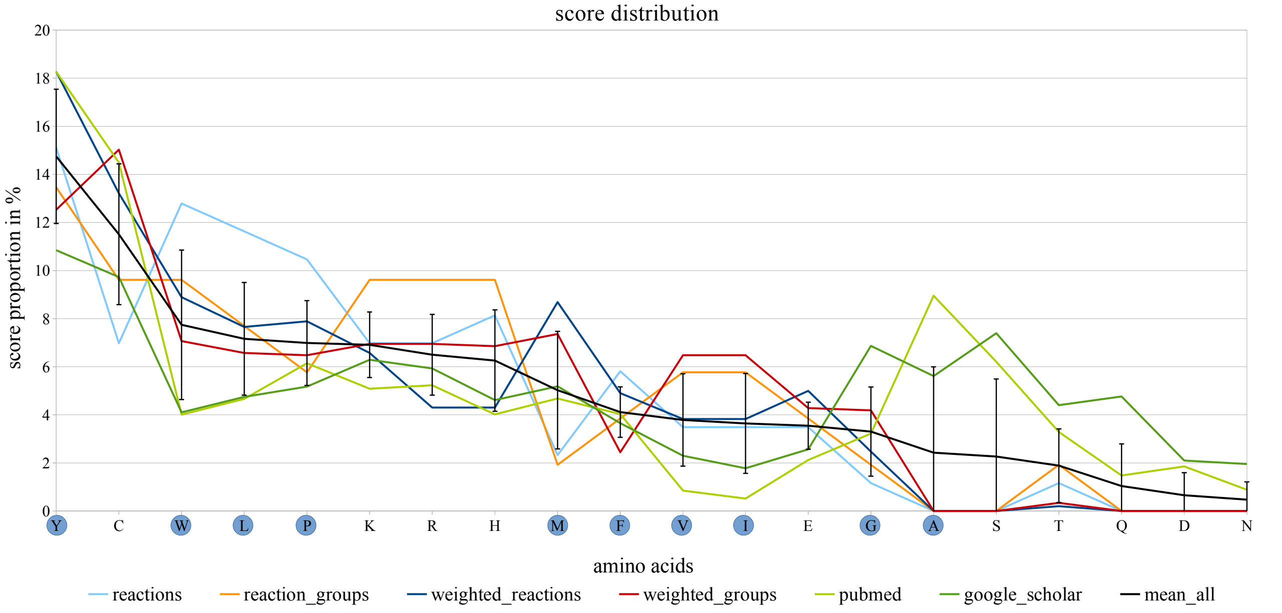

Suppl. Figure 1: Score distribution from Fichtner et al. (2019) sorted by score. The amino acids classified by us as hydrophobic are marked with blue circles. This diagram was created with LibreOffice Calc version 6.4.6.2.

2. Spatial accessibility of secondary structures

Suppl. Table 2: Summary of secondary structures and possible spatial susceptibility. The spatial features are derived from the chemical structures.

**Secondary structure**	**Spatial accessibility**
α-, 3_{10}-, π-Helix	backbone lies covered
Collagen-helix	AAs lie partly covered (periodic)
β-Helix	backbone only attackable from one side
β-Sheet	completely open
β-Turn	backbone very lightly covered
Random coil	variable accessibility

3. Data preparation

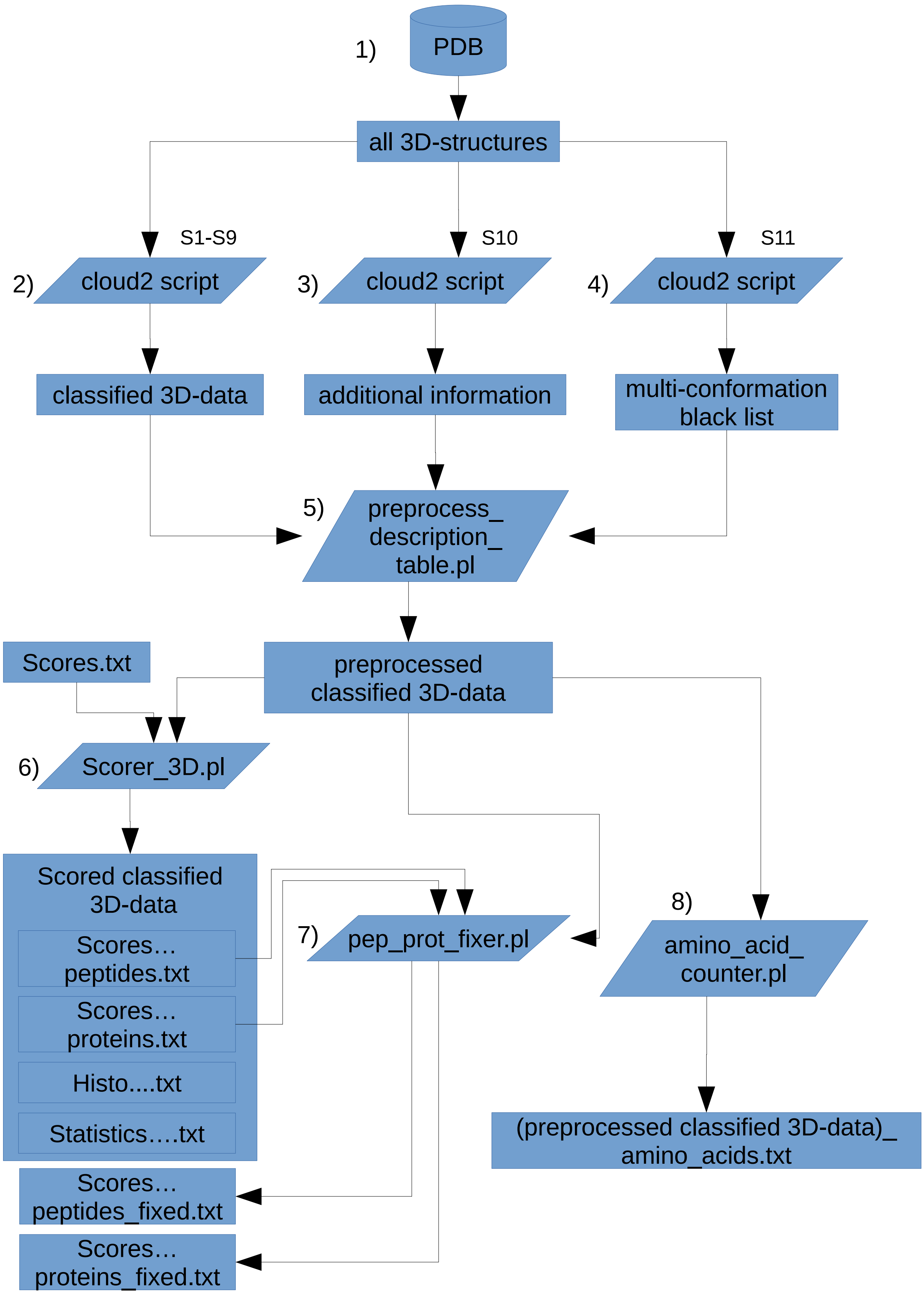

Suppl. Figure 2: Flowchart of the whole process of generating the scores and counting of the amino acids. This flowchart was created by MF with LibreOffice Impress version 6.4.6.2.

1) The spatial data was downloaded from the Protein Data Bank (PDB) (ftp.wwpdb.org/pub/pdb/data/structures/all/pdb; 02.05.2018).

2) The scripts #1 – #9 (see Appendix) were used in the program Cloud2 Version 14.3.20 (Heiko Stark, Jena, Germany, URL: https://starkrats.de) to extract the respective amino acids (AA). This resulted in the different sequence files (‘all’, ‘core’, ‘hull’, ...) modified with the respective spatial information. This leads to 9 different data sets, but for simplicity it is summarized here as classified 3D-data. That means the following steps have to be done separately for every data set.

3)The script #10 was used to retrieve additional information like descriptions and organisms out of the structure files to include them later.

4) Multi-conformational entries could not be handled at this point, that is why script #11 was used to make a list of all multi-conformational IDs to exclude these sequences later.

Step #2 to #4 can also be done in one, but due to the development process we came to this modularised approach which gives the possibility to alter one aspect without having to recalculate all the spatial data.

5) The perl (v5.22.1) program ‘preprocess_description_table.pl’ combines the gathered information from step #2 to #4. It adds the additional information to the headers of the calculated sequences and removes the sequences with the IDs from the multi-conformation black list. The resulting files now have the ending ‘..._descr...’ added to them but are here listed as preprocessed classified 3D-data.

6) The program ‘Scorer_3D.pl’ is a modification of the scoring program from Fichtner et al. (2019)[5]. It leaves out the GO-annotation. It also uses the scores from the table in ‘Scores.txt’. For every data set a peptide and protein score file is created as well as a histogram and a statistics file. Note that with this approach, another step follows that will very likely change the final statistics (step #7). See also supplement of Fichtner et al. (2019)[5] for further details.

7) Because the sequences are reduced in step #2 (except for the data set ‘all’) the allocation to peptides and proteins is not working as intended in step #6. That means proteins can be degraded to peptides if they fall under the threshold of 100 AAs. The program ‘pep_prot_fixer.pl’ is there to reallocate the false positive peptides. It takes the peptide and the protein score file, compares them with the preprocessed classified 3D-data and does the necessary arrangements. It also removes all entries with an ‘X’-amount higher than the chosen threshold. In our case we decided to use 0.05 as a default value. The created score files have the ending ‘..._fixed...’ and are the final result in the chain. Additionally, the program ‘no_length_0.pl’ can be applied to get a copy without sequences of length 0, that can result especially in peptides due to the differentiation between core and hull (e.g. if there is no core.

8) An additional consideration is made in step #8 out of the ‘preprocessed classified 3D-data’. The program ‘amino_acid_counter.pl’ is counting the occurrence of all characters. The results are discussed in our paper and in Suppl. 3 there are additional diagrams for this approach.

4. Extended Graham Scan

For our calculations we used the Graham Scan (https://en.wikipedia.org/wiki/Graham_scan). With the algorithm the convex hull of a finite set of points in 2D can be calculated.
In a second step we check which line connecting two atoms in the hull is longer than the minimal distance (6 Å or 7 Å). If so, the line is replaced by two or more lines connecting the two atoms with the closest indented node(s). Finally this results in the concave hull. This simplification is generalized to sectional planes along the three spatial axes to identify all surface-associated atoms. In this way, we obtain the concave hull, but only for topologies with genus zero (i.e. without holes).
The code can be found under https://github.com/heikostark/Projekte/blob/cloud2/mesh.inc
Line 251 to 704 contains the code for the extended Graham Scan.

5. Statistics of the influence of the spatial protein structure on the susceptibility

Note that these are not the statistics from the statistics file (see Suppl.3, #step 6). Since the statistics are changed by a step afterwards, these values have been obtained with an R script that can be found in the supplementary data. It is called 'boxplot_proteins' and contains also the code for the plots from Fig. 2.

For 6 Å:

Suppl. Table 3: Statistics of the influence of the spatial peptide structure WP=whole protein, PC=protein core, PS=protein shell, SSC= shell side chain, SBB= shell backbone) on the susceptibility of peptides for a cavity of 6 Å.

Peptides	Min	Q1	Median	Q2	Max	Mean	SD
WP	0.76	4.13	4.53	4.99	13.15	4.61	0.7
PC	0	4.14	4.83	5.78	12.42	5.11	1.53
PS	0.76	4.01	4.39	4.82	13.15	4.45	0.7
SSC	0	4.01	4.41	4.86	13.15	4.47	0.75
SBB	0.76	3.61	4.08	4.6	12.29	4.16	0.86

Suppl. Table 4: Statistics of the influence of the spatial protein structure (WP=whole protein, PC=protein core, PS=protein shell, SSC= shell side chain, SBB= shell backbone) on the susceptibility of proteins for a cavity of 6 Å.

Proteins	Min	Q1	Median	Q2	Max	Mean	SD
WP	2	4.36	4.5	4.59	8.59	4.44	0.38
PC	1.26	4.19	4.65	4.88	10.38	4.63	0.54
PS	1.15	3.9	4.15	4.42	8.53	4.18	0.42
SSC	0	3.92	4.22	4.5	14.75	4.22	0.46
SBB	0.14	3.71	3.96	4.36	13.12	4.04	0.5

For 7 Å:

Suppl. Table 5: Statistics of the influence of the spatial peptide structure (WP=whole protein, PC=protein core, PS=protein shell, SSC= shell side chain, SBB= shell backbone) on the susceptibility of peptides for a cavity of 7 Å.

Peptides	Min	Q1	Median	Q2	Max	Mean	SD
WP	0.76	4.13	4.53	4.99	13.15	4.61	0.7
PC	0.47	4.12	4.79	5.67	12.32	5	1.41
PS	0.76	3.99	4.39	4.81	13.15	4.45	0.7
SSC	0	4	4.41	4.85	13.15	4.46	0.75
SBB	0.7	3.53	4.02	4.59	12.91	4.11	0.93

Suppl. Table 6: Statistics of the influence of the spatial protein structure (WP=whole protein, PC=protein core, PS=protein shell, SSC= shell side chain, SBB= shell backbone) on the susceptibility of proteins for a cavity of 7 Å.

Proteins	Min	Q1	Median	Q2	Max	Mean	SD
WP	2	4.36	4.5	4.59	8.59	4.44	0.38
PC	1.53	4.18	4.66	4.9	9.25	4.62	0.51
PS	0.63	3.88	4.13	4.44	8.33	4.19	0.44
SSC	0	3.87	4.17	4.49	14.75	4.21	0.5
SBB	0.11	3.74	4.05	4.37	14.75	4.07	0.62

6. Example Comparison with and without water and ligands

We have taken the file for the protein human oxyhaemoglobin (PDB entry: 1hho) which is given in the PDB with water and ligands. For the comparison we have analyzed this protein (which also shown in the Figure 1 in the main text) with and without these additional molecules (see Suppl. Fig.x)

Our analysis showed, that the protein shell now contains 148 amino acids in the presence of water and ligands and 167 in their absence. That means 19 amino acids are no longer shielded. These AAs show a spatially heterogeneous distribution. However, the difference between core and shell remains (see http://damage.stark-jena.de).

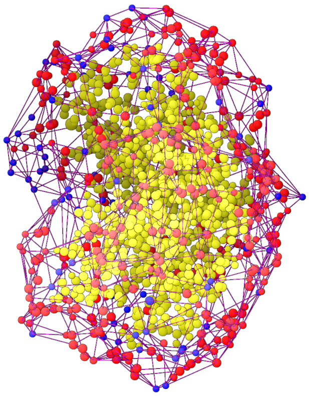

a)

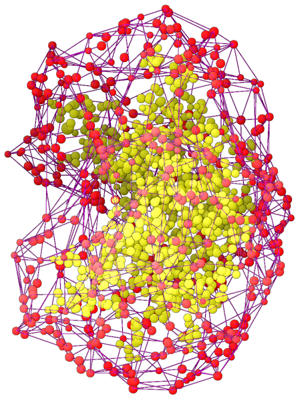

b)

Suppl. Fig. 3: Representation of the amino acids with (a) and without (b) water/ligand components of the protein oxyhaemoglobin (PDB entry: pdb1hho). A dot represents the averaged centre of an amino acid (red when in the protein shell (PS), yellow in the protein core (PC)) or water molecule/ ligands (blue). Red lines show the cross-linking by the surface calculation, by which the surface (concave hull) is defined. All unlinked dots are assigned to the PC. This graphic was created with Cloud2 version 15.7.22 (https://starkrats.de).

7. Further amino acid distribution visualizations

In the PDB data the information is resolved on the level of atoms which are allocated to different AAs. There are also other entries than the 20 standard AAs listed. This includes next to the very rare selenocysteine ('U'; only 191 occurrences) and pyrrolysine ('O', only 2 occurrences) also unknown positions ('X'). Even other molecules like ligands or water on the protein surface are listed. These entries are put together in one 'X' at the end of the sequence by cloud2. For the creation of the diagrams only the information for the 20 standard AAs was considered. That means especially in the calculation 'X's for example were not considered.
For a closer look at AA distributions Suppl. Fig. 3 shows the distribution of all standard AAs over all proteins we analyzed from PDB.

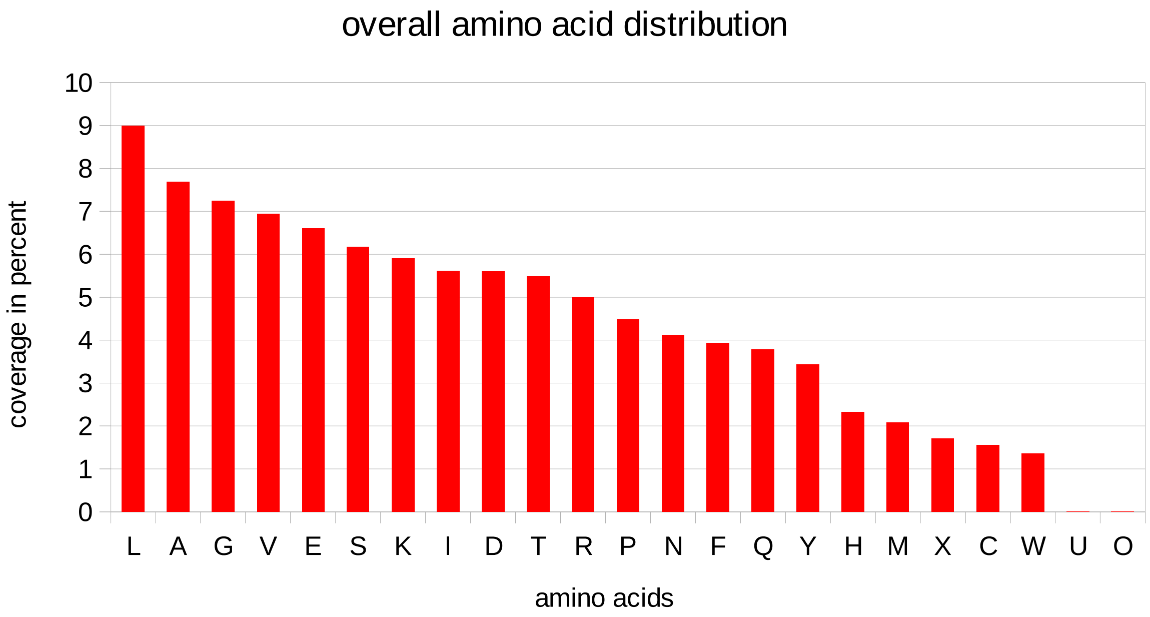

Suppl. Figure 4: Normalized amino acid distribution for all proteins with structure information from PDB. This diagram was created with LibreOffice Calc version 6.4.6.2.

In the following some normalized versions for the AA distributions with different focuses are shown. Suppl. Fig. 4 shows how the AAs distribute over protein shell (PS) and core (PC). Here a normalization is shown where the real values of an AA for PS and PC are divided by the sum of all values for that AA. In addition, it was changed into percent where PS+PC for every AAs equals 100 %. This shows the distribution of the respective AA over PS and PC. As expected the PC dominates most of the entries which is because the complete data set of PDB contains many bigger proteins. With increasing size the share of AAs considered to be in the core increases in general

much more than in the PS which leads to the PC containing most of the AAs. That is why another normalization step was made.

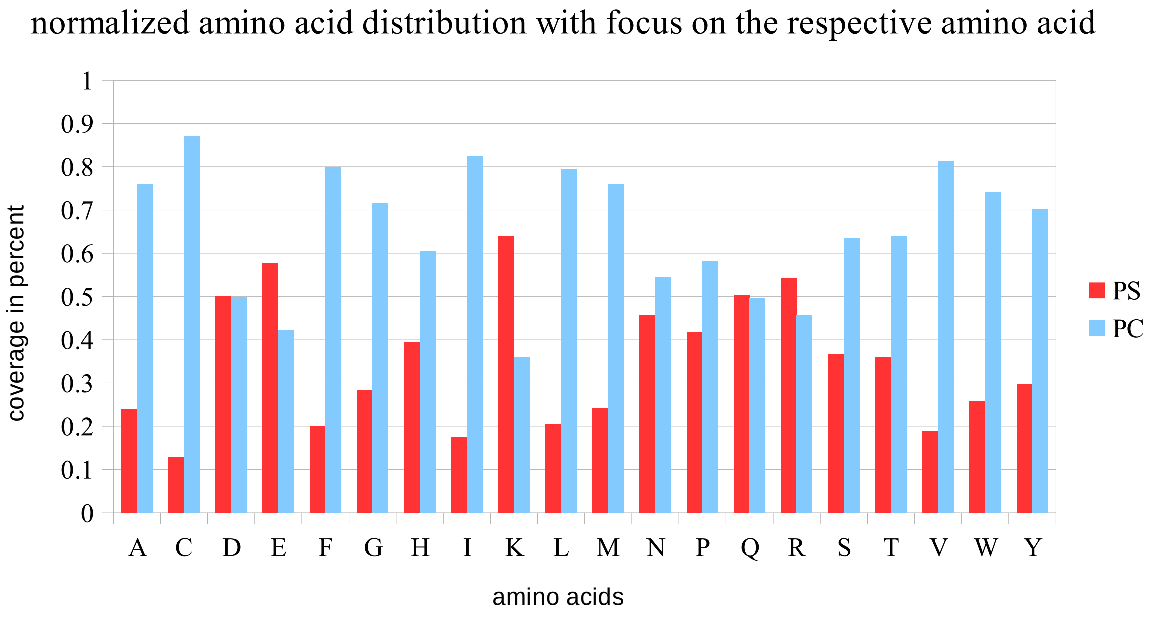

Suppl. Figure 5: Normalized amino acid distribution with focus on the respective amino acid. The protein shell (PS) and core (PC) value for every amino acid add up to 100 %. For example for all alanine over all proteins 24 % distribute on the PS and 76 % on the PC. This diagram was created with LibreOffice Calc version 6.4.6.2.

The next figure (Suppl. Fig. 5) focuses on the other direction. Here, it is shown how the PS and PC distribute over all the AAs. Therefore, the real values of an AA for PS and PC are divided by the sum of all respective PS or PC values. In addition, it was changed into percent where all the values for PS/PC together equal 100 %. This shows the distribution of the shell or the core over all AAs. This normalization is the best to compare the PS with the PC proportionally.

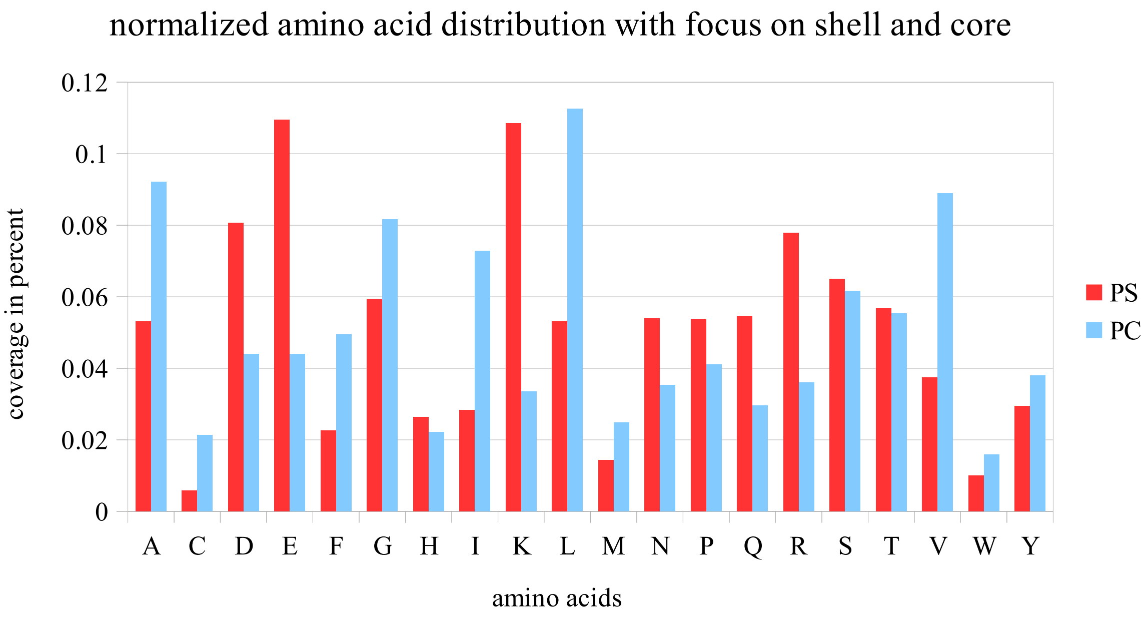

Suppl. Figure 6: Normalized amino acid distribution with focus on protein shell (PS) and core (PC). All red columns together add up to 100 % (PS) and all blue columns together add up to 100 % (PC). For example for all amino acids on the PS about 5 % are alanine and of all amino acids in the PC about 9 % are alanine. This diagram was created with LibreOffice Calc version 6.4.6.2.

8. Further comparison of protein shell and core properties

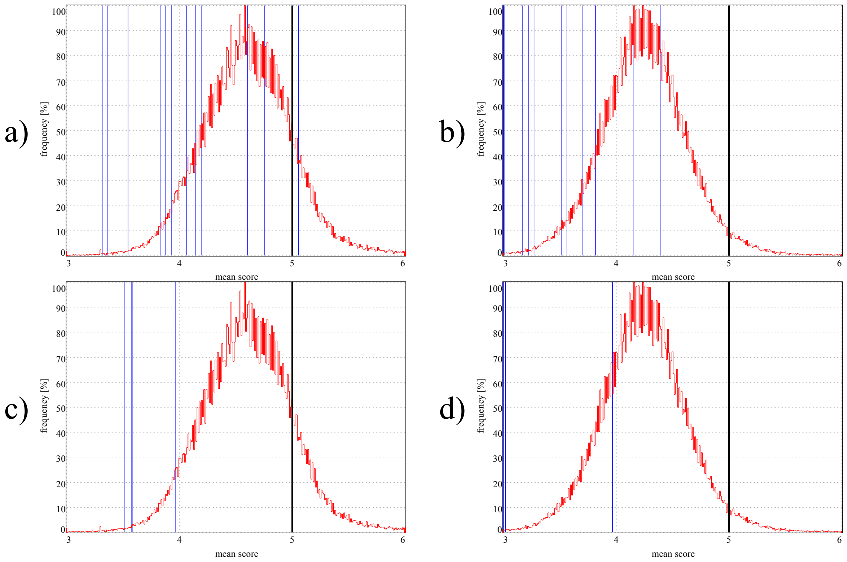

Suppl. Figure 7: Diagrams of the mean score of protein core (PC) and protein shell (PS) of flaggellin and spidroin. a) PC flagellin, b) PS flagellin, c) PC spidroin, d) PS spidroin. The red graph shows the histogram of all proteins and the vertical blue lines indicate the position of the protein entries. The black line indicates the mean score of a random artificial protein. These graphics were created with Enzyme2 version 9.7.22 (https://starkrats.de).

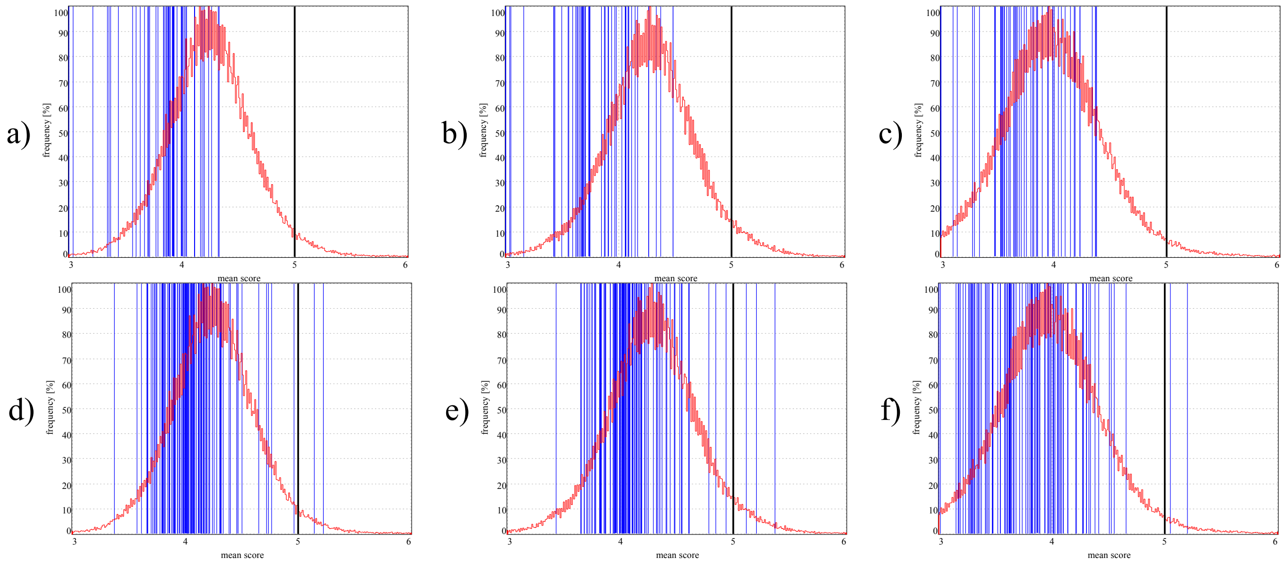

Suppl. Figure 8: Diagrams of the mean score of shell types protein shell (PS), shell backbone (SBB), shell side chain (SSC) for antifreeze and heat shock. a) PS antifreeze, b) SBB antifreeze, c) SSC antifreeze, d) PS heat shock e) SBB heat shock f) SSC heat shock. The red graph shows the histogram of all proteins and the vertical blue lines indicate the position of the protein entries. The black line indicates the mean score of a random artificial protein. These graphics were created with Enzyme2 version 9.7.22 (https://starkrats.de).

For more examples we refer to our website http://damage.stark-jena.de.

9. Further comparison of enzyme properties

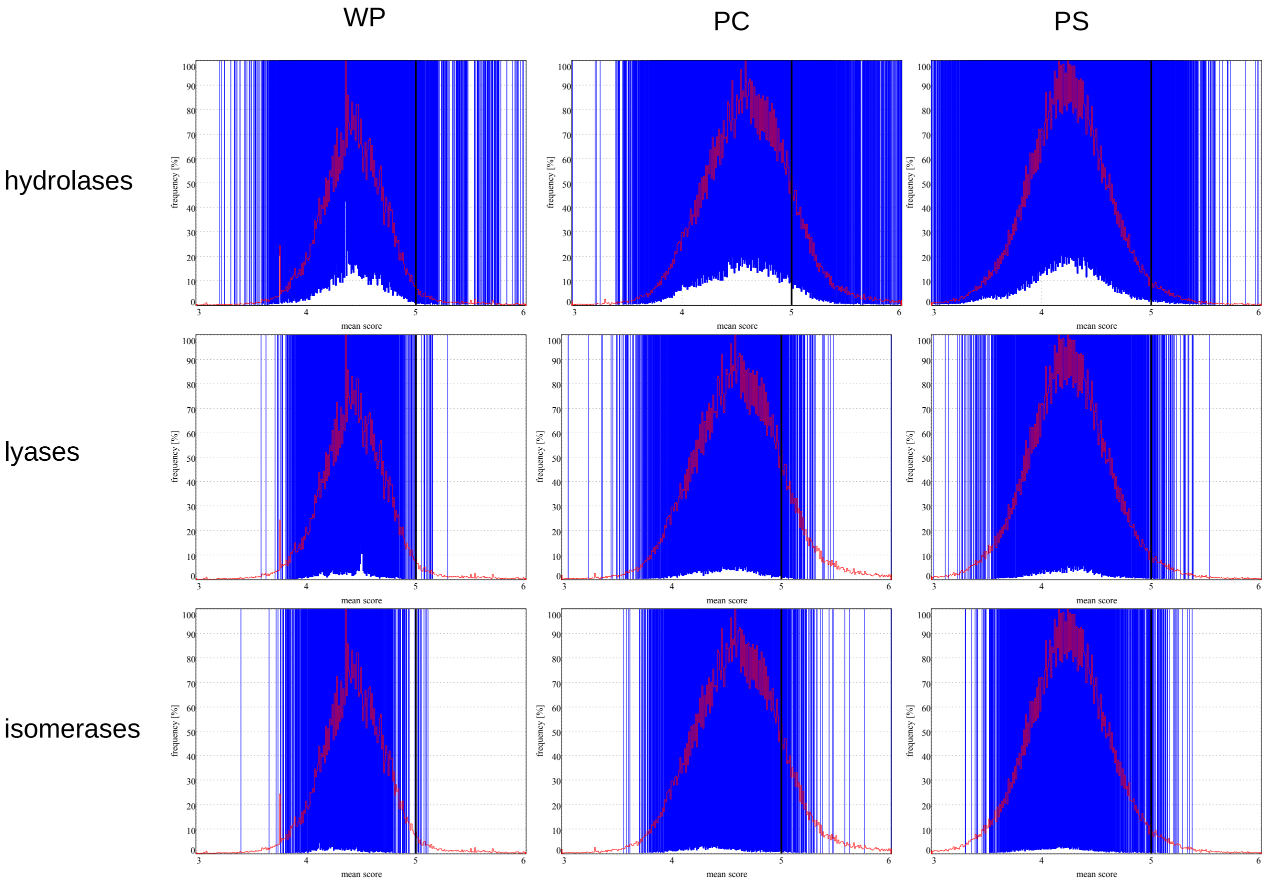

Suppl. Figure 9: Diagrams of the mean score of whole protein (WP), protein core (PC) and protein shell (PS) of hydrolases (EC:03), lyases (EC:04) and isomerases (EC:05). The red graph shows the histogram of all proteins and the vertical blue lines indicate the position of the protein entries. The black line indicates the mean score of a random artificial protein. Created with Enzyme2 version 9.7.22 (https://starkrats.de).

For more examples we refer to our website http://damage.stark-jena.de.

10. Comparison between organisms

Our analysis revealed visible differences between organisms from different kingdoms (Suppl. Fig. 9). Bacteria show more unsusceptible proteins than other organisms, while animals show more susceptible proteins. The plants, which seem neither susceptible nor unsusceptible, occupy a special position.

Calculation and normalization

The calculation for the Supplement Figures 9 and 10 were calculated in two cases. The two cases take into account the difference between the relation of the mean score for the protein shell (PS) and the protein core (PC). In case 2 a normalization has been made, to show all values in the range of 1 to 2 and with that overall in the range of 0 to 2.

case 1 mean score PS < mean score PC : mean score PS / mean score PC
case 2 mean score PS > mean score PC : 1+(1- (mean score PC / mean score PS))

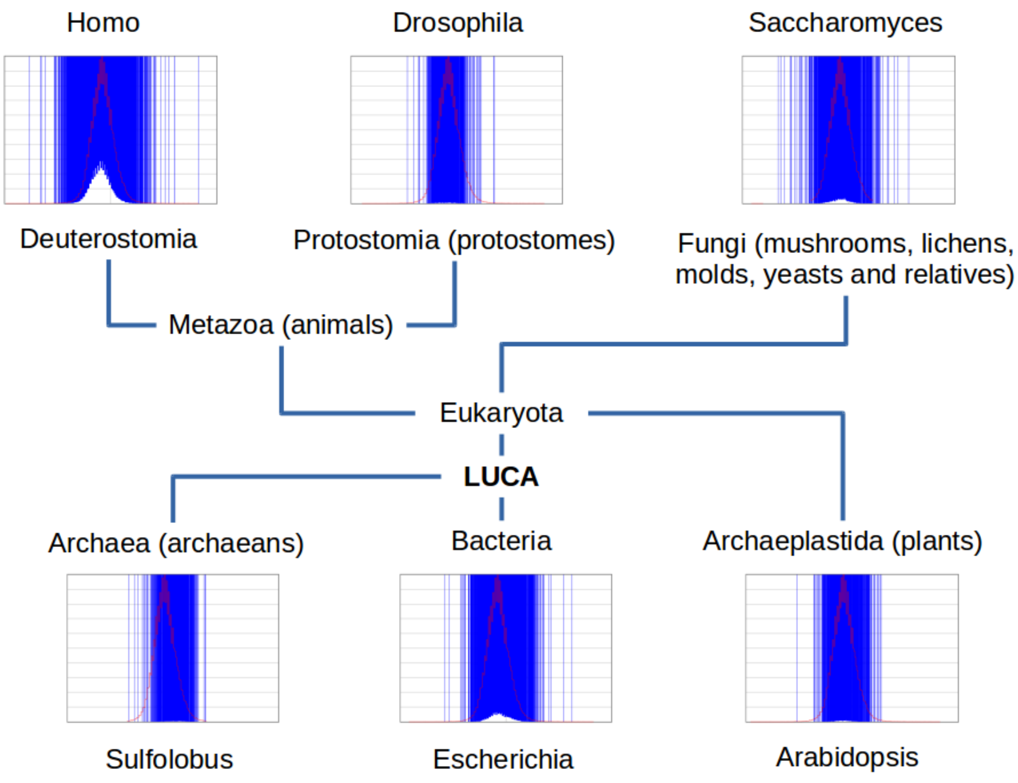

Suppl. Figure 10: Cladogram of the genera *Sulfolobus, Escherichia, Arabidopsis, Saccharomyces, Drosophila and Homo*. The leafs/clades show the distribution of the peptides and proteins with regard to the difference between protein core (PC) and shell (PS) (see difference calculation). These graphics were created with Enzyme2 version 9.7.22 (https://starkrats.de) and LibreOffice Impress version 6.4.6.2.

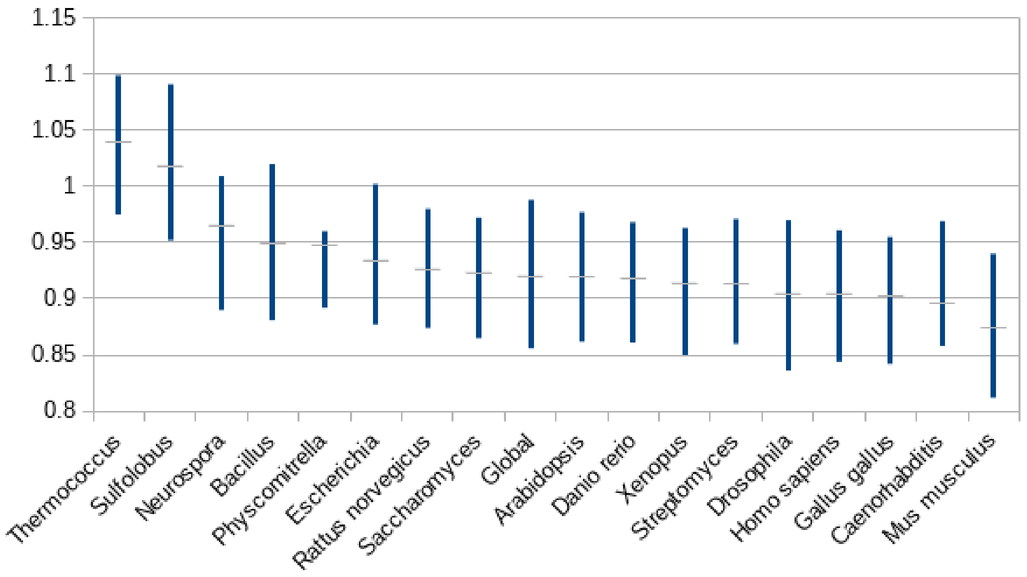

Suppl. Figure 11: Sorted representation of the organisms with regard to the susceptibility between protein shell (PS) and protein core (PC) (see difference calculation). Values above 1 mean a more susceptible PS compared to the PC and values below 1 mean a more susceptible PC compared to the PS. This diagram was created with LibreOffice Calc version 6.4.6.2.

A more detailed analysis of the distribution shows differences between organisms in the susceptibilities in PS and PC. These clearly occur in the *Archea*, since on average they have a more susceptible PS than the PC (Suppl. Fig. 10). It is noticeable that *Rattus norvegicus* and *Mus musculus* differ greatly from their closest relatives (this may be due to the fact that these are experimental animals and therefore certain protein classes are overrepresented). Otherwise, the organisms are divided into unicellular and multicellular groups.

For more examples we refer to our website http://damage.stark-jena.de.

11. Top and Bottom 100

For the purpose of analysis and as an overview, we made a list for every protein part, which we distinguished in our work, with the top 100 and bottom 100 entries of the susceptibility calculation. The file is called “TopBot100.ods”. These entries also contain the information for the organism and between the lists we counted the occurrence of some specific organisms. So you can see and compare which organisms rank relatively high or low in the respective protein part.

12. Appendix

Script 1: all

```
io.files *.ent.gz
io.files.sort

for io.files.length

   new.all
   set.red
   filename := io.workdir / io.files.getdir / io.files.getfile
```

```
    import.pdb.atom filename

    filename := io.workdir / io.files.getdir / 'result_all.txt'
    append.aminoacids filename

    io.files.next

  end
```

Script 2: core_6

```
io.files *.ent.gz
io.files.sort

for io.files.length

    new.all
    set.red
    filename := io.workdir / io.files.getdir / io.files.getfile
    import.pdb.atom filename
    edit.name.to.unique
    set.ref.green
    bool.copy.layer

    set.ref.purple
    mesh.point.hull.x <0, 0, 0> 3 6 0
    mesh.point.hull.y <0, 0, 0> 3 6 0
    mesh.point.hull.z <0, 0, 0> 3 6 0
    set.purple
    edit.erase.duplicates

  // mask aminoacids
    set.red
    set.ref.purple
    bool.inside.circle
    bool.mask.points

  // mask core aminoacids
    set.green
    set.ref.red
    bool.outside.circle
    bool.mask.objects

    filename := io.workdir / io.files.getdir / 'result_core_6.txt'
    append.aminoacids filename

    io.files.next

  end
```

Script 3: core_7

```
  io.files *.ent.gz
  io.files.sort

  for io.files.length
```

```
    new.all
    set.red
    filename := io.workdir / io.files.getdir / io.files.getfile
    import.pdb.atom filename
    edit.name.to.unique
    set.ref.green
    bool.copy.layer

    set.ref.purple
    mesh.point.hull.x <0, 0, 0> 3.5 7 0
    mesh.point.hull.y <0, 0, 0> 3.5 7 0
    mesh.point.hull.z <0, 0, 0> 3.5 7 0
    set.purple
    edit.erase.duplicates

  // mask aminoacids
    set.red
    set.ref.purple
    bool.inside.circle
    bool.mask.points

  // mask core aminoacids
    set.green
    set.ref.red
    bool.outside.circle
    bool.mask.objects

    filename := io.workdir / io.files.getdir / 'result_core_7.txt'
    append.aminoacids filename

    io.files.next

  end
```

Script 4: hull_6

```
  io.files *.ent.gz
  io.files.sort

  for io.files.length

    new.all
    set.red
    filename := io.workdir / io.files.getdir / io.files.getfile
    import.pdb.atom filename

    set.ref.purple
    mesh.point.hull.x <0, 0, 0> 3 6 0
    mesh.point.hull.y <0, 0, 0> 3 6 0
    mesh.point.hull.z <0, 0, 0> 3 6 0
    set.purple
    edit.erase.duplicates
    set.red
    set.ref.purple
    bool.inside.circle
    bool.mask.points

    filename := io.workdir / io.files.getdir / 'result_hull_6.txt'
    append.aminoacids filename
```

```
    io.files.next

end
```

Script 5: hull_7

```
io.files *.ent.gz
io.files.sort

for io.files.length

    new.all
    set.red
    filename := io.workdir / io.files.getdir / io.files.getfile
    import.pdb.atom filename

    set.ref.purple
    mesh.point.hull.x <0, 0, 0> 3.5 7 0
    mesh.point.hull.y <0, 0, 0> 3.5 7 0
    mesh.point.hull.z <0, 0, 0> 3.5 7 0
    set.purple
    edit.erase.duplicates
    set.red
    set.ref.purple
    bool.inside.circle
    bool.mask.points

    filename := io.workdir / io.files.getdir / 'result_hull_7.txt'
    append.aminoacids filename

    io.files.next

end
```

Script 6: hull-backbone_6

```
io.files *.ent.gz
io.files.sort

for io.files.length

    new.all
    set.red
    filename := io.workdir / io.files.getdir / io.files.getfile
    import.pdb.backbone filename

    set.ref.purple
    mesh.point.hull.x <0, 0, 0> 3 6 0
    mesh.point.hull.y <0, 0, 0> 3 6 0
    mesh.point.hull.z <0, 0, 0> 3 6 0
    set.purple
    edit.erase.duplicates
    set.red
    set.ref.purple
    bool.inside.circle
    bool.mask.points
    bool.erase.line.materials -100 0

    filename := io.workdir / io.files.getdir / 'result_hull-backbone_6.txt'
```

```
        append.aminoacids filename

        io.files.next

    end
```

Script 7: hull-backbone_7

```
    io.files *.ent.gz
    io.files.sort

    for io.files.length

        new.all
        set.red
        filename := io.workdir / io.files.getdir / io.files.getfile
        import.pdb.backbone filename

        set.ref.purple
        mesh.point.hull.x <0, 0, 0> 3.5 7 0
        mesh.point.hull.y <0, 0, 0> 3.5 7 0
        mesh.point.hull.z <0, 0, 0> 3.5 7 0
        set.purple
        edit.erase.duplicates
        set.red
        set.ref.purple
        bool.inside.circle
        bool.mask.points
        bool.erase.line.materials -100 0

        filename := io.workdir / io.files.getdir / 'result_hull-backbone_7.txt'
        append.aminoacids filename

        io.files.next

    end
```

Script 8: hull-side_chain_6

```
    io.files *.ent.gz
    io.files.sort

    for io.files.length

        new.all
        set.red
        filename := io.workdir / io.files.getdir / io.files.getfile
        import.pdb.backbone filename

        set.ref.purple
        mesh.point.hull.x <0, 0, 0> 3 6 0
        mesh.point.hull.y <0, 0, 0> 3 6 0
        mesh.point.hull.z <0, 0, 0> 3 6 0
        set.purple
```

```
    edit.erase.duplicates
    set.red
    set.ref.purple
    bool.inside.circle
    bool.mask.points
    bool.erase.line.materials 0 100

    filename := io.workdir / io.files.getdir / 'result_hull-side_chain_6.txt'
    append.aminoacids filename

    io.files.next

end
```

Script 9: hull-side_chain_7

```
io.files *.ent.gz
io.files.sort

for io.files.length

    new.all
    set.red
    filename := io.workdir / io.files.getdir / io.files.getfile
    import.pdb.backbone filename

    set.ref.purple
    mesh.point.hull.x <0, 0, 0> 3.5 7 0
    mesh.point.hull.y <0, 0, 0> 3.5 7 0
    mesh.point.hull.z <0, 0, 0> 3.5 7 0
    set.purple
    edit.erase.duplicates
    set.red
    set.ref.purple
    bool.inside.circle
    bool.mask.points
    bool.erase.line.materials 0 100

    filename := io.workdir / io.files.getdir / 'result_hull-side_chain_7.txt'
    append.aminoacids filename

    io.files.next

end
```

Script 10: table

```
io.files *.ent.gz
io.files.sort

tab := '	'

store '../table.pdb'
@ 'ID' tab '3DID' tab 'EC' tab 'CLASS' tab 'TITLE' tab 'ORGANISM'
end

for io.files.length

    new.all
```

```
    filename := io.workdir / io.files.getdir / io.files.getfile
    import.pdb.atom filename

    filename := '../table.pdb'
    append filename
@ io.files.getname tab pdb.id tab pdb.ec tab pdb.class tab pdb.title tab pdb.organism
    end

    io.files.next

end
```

Script 11: multimodels

```
io.dirs.complete * // load all sub dirs into a dir list

io.files *.ent.gz

output.store "models.txt"

for io.files.length // for all files
        filename := io.workdir / io.files.getdir / io.files.getfile
        bool := text.find filename "MODEL        2" "MODEL        3"
        if bool
        then print filename
        io.files.next
end

end
```

13. References

1. Kästle, M. & Grune, T. Protein oxidative modification in the aging organism and the role of the ubiquitin proteasomal system. *Curr. Pharm. Des.* **17**, 4007–4022 (2011).
2. Voet, D. & Voet, J. G. *Biochemistry*. (Wiley, 2004).
3. Lesk, A. *Introduction to Bioinformatics*. (OUP Oxford, 2013).
4. Berg, J. M., Stryer, L. & Tymoczko, J. L. *Stryer Biochemie*. (Springer-Verlag, 2015).
5. Fichtner, M., Schuster, S. & Stark, H. Determination of scoring functions for protein damage susceptibility. *BioSystems* 104035 (2019) doi:10.1016/j.biosystems.2019.104035.
